# Supplementary material for: Fluids as primary carriers of sulphur and copper in magmatic assimilation
Source: Nat Commun. 2021 Nov 16;12:6609. doi: 10.1038/s41467-021-26969-3 (PMC8595724; doi:10.1038/s41467-021-26969-3)
Supplement: Supplementary file 1 — Supplementary Information [file 41467_2021_26969_MOESM1_ESM.pdf]

SUPPLEMENTARY INFORMATION TO MANUSCRIPT "*FLUIDS AS PRIMARY CARRIERS OF SULPHUR AND COPPER IN MAGMATIC ASSIMILATION*"

Virtanen et al.

SUPPLEMENTARY NOTES

Location of the starting material sample

The location of the starting material sample is shown in the geological map in Fig. S1. The sample comes from a Virginia Formation drill core that was drilled more than 10 km away from the Duluth Complex, Minnesota (Fig. S1). The locations of the Partridge River Intrusion and South Kawishiwi Intrusion as well as the cross-section referred to in Fig. 7 of the main manuscript are also indicated in the map (Fig. S1).

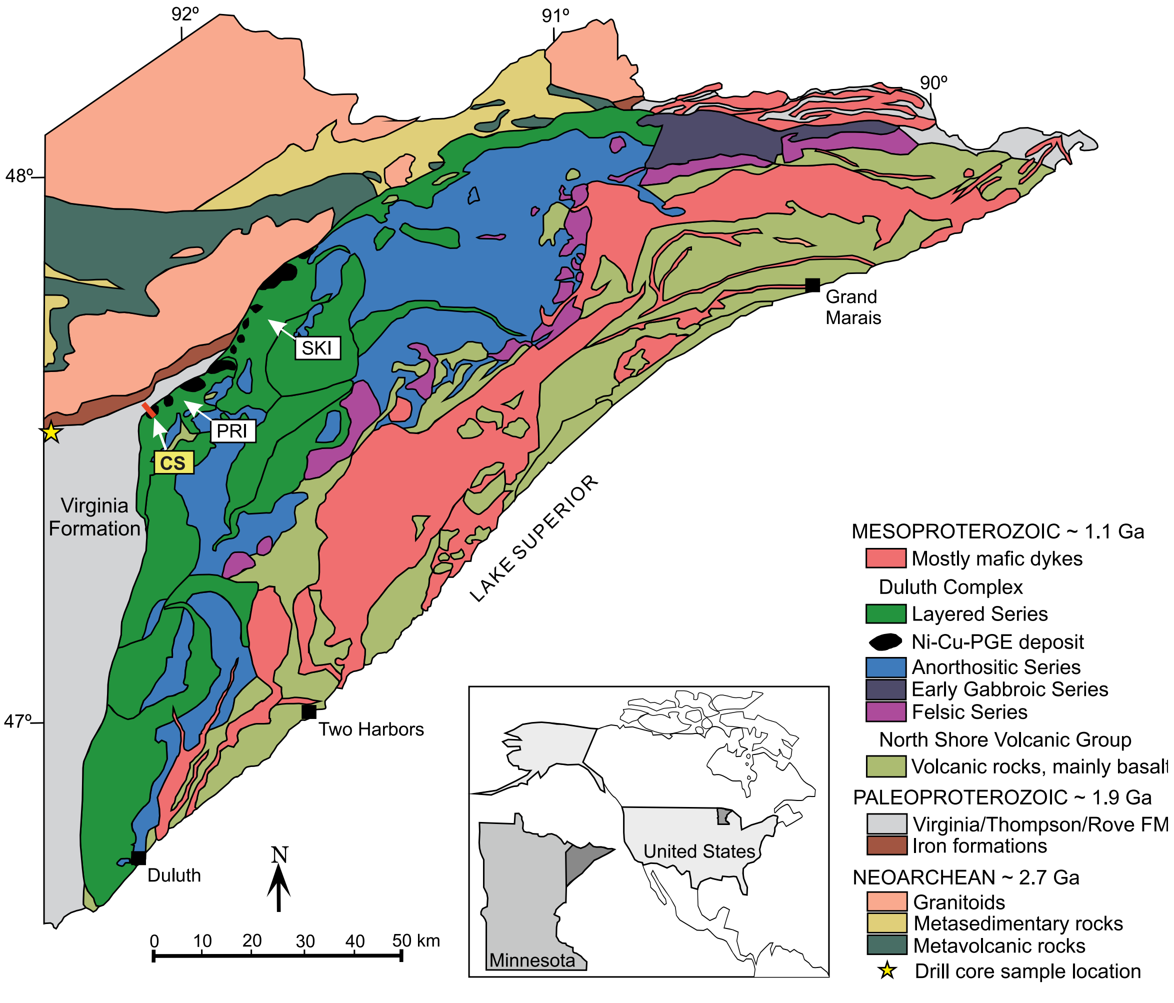

Figure S1. Geological map of the Duluth Complex, Minnesota and surrounding rocks. The location of the drill core MDDP2 is indicated with a star. The abbreviation PRI stands for Partridge River Intrusion, SKI for South Kawishiwi Intrusion, and CS for the cross-section in the Fig. 7. Modified after Bénko et al.[17].

Failed experiments

For the benefit of those researchers, who plan to conduct experiments similar to ours, we shortly describe the failed experiments here. We performed experiments at 1100 °C (200 MPa, 48 h), but in those experiments the sulphides reacted with the capsule material (in these experiments capsules were $Au_{80}Pd_{20}$, as 1100 °C is close to the $Au_{90}Pd_{10}$ melting point), as evidenced by the disappearance of Cu from most of the sulphide droplets. The few sulphide droplets that contained Cu also contained Pd. Palladium was selectively scavenged from the capsule walls, because Au was not detected in the sulphide droplets. We interpret that Cu alloyed with Pd and hence the results of those experiments are not included in the main article.

We tried to prevent the run charge from reacting with the capsule material by performing experiments with an inner graphite capsule within the Au-Pd capsules, but the sample material experienced significant fluid loss, as the graphite capsules were permeable to the fluid. For this reason, we do not recommend the usage of graphite capsules in fluid-saturated experiments. Additionally, we observed the Cu-Pd alloys within the sample sealed in the graphite capsules at 1100 °C. We state this information here as it may be valuable to researchers studying the fluid transport of Pd.

SUPPLEMENTARY METHODS

Identification of graphite and ferrogedrite using Raman Spectroscopy

After the reported measurements with the FE-SEM, the capsules (then mounted in epoxy and ground in half) from the 700 °C, 800 °C, 900 °C, and 1000 °C experiments were prepared for Raman spectroscopy. These analyses were needed to identify graphite and ferrogedrite grains, which are too small for FE-SEM EDS measurements. In order to enable the analysis of the sample without possible surface defects caused by polishing and contamination from carbon coating, we had to further saw the capsules into two quarters and remove sample material from the capsules

(Fig. S2). The capsules were sawed using a Struers Accutom-10 precision saw equipped with a diamond cut-off wheel (diameter 76 mm, thickness 0.15 mm) at the Department of Physics, University of Helsinki. The quarter capsules were removed from the epoxy and the sample material was removed by gently bending the capsule walls with pliers (Fig. S2). The Raman spectroscopy measurements were conducted on the sample surface that was in contact with the capsule (Fig. S2).

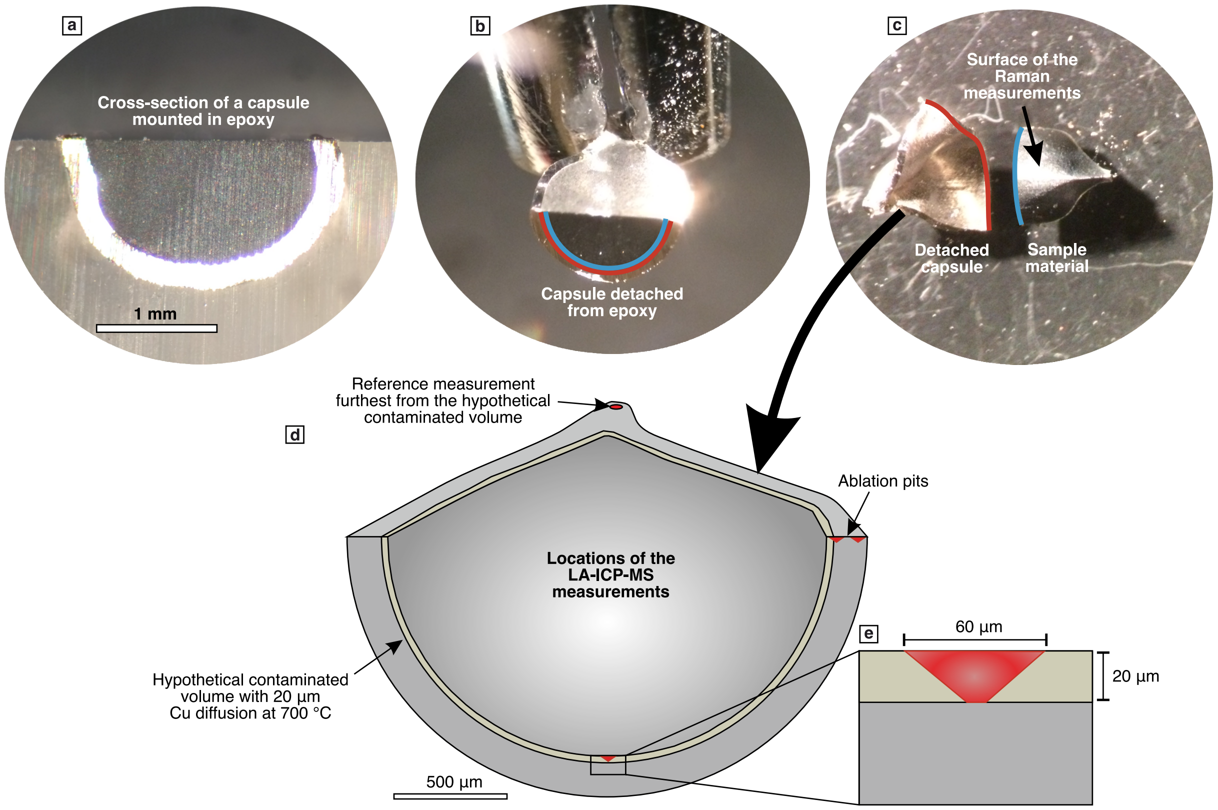

Figure S2. A schematic presentation of the capsule preparations for the laser ablation inductively coupled plasma mass spectrometry (LA-ICP-MS) and Raman spectrometry measurements. The quartered sample capsule (**a**) was removed from the epoxy with pliers (**b**) and then the capsule walls were bent to remove the sample material (**c**). The red line in the top center and top right indicate the same edge on the sample material and capsule (notice the bent walls on the **c**). The Raman spectroscopy measurements were conducted on the surface in contact with the capsule to avoid polishing defects and contamination from carbon coating (**c**). The schematic shows an empty quarter capsule and the red ablation pits indicate typical locations of the LA-ICP-MS measurements (**d**). The enlargement (**e**) shows the shape and size of the ablation pit as approximated with reflected light microscopy. The hypothetical contaminated volume (20 µm Cu diffusion into the capsule wall) was calculated using the Arrhenius law (see the section sulphur and copper distribution in the 700 °C experiment).

Raman spectra of graphitic carbon were identified in the 700 °C, 800 °C, 900 °C, and 1000 °C experiment products. Despite of the high background signal, two Raman shift peaks at ~1350

cm^{-1} and ~1600 cm^{-1} are clearly present (Fig. S3). The graphite standard shows a characteristic Raman shift peak at 1580 cm^{-1} (Fig. S3). The presence of the structural disorder peaks *D1* and *D2* indicates that the graphitic carbons in our samples are more disordered than the pure graphite standard, which is common to experimentally produced graphite[1–3].

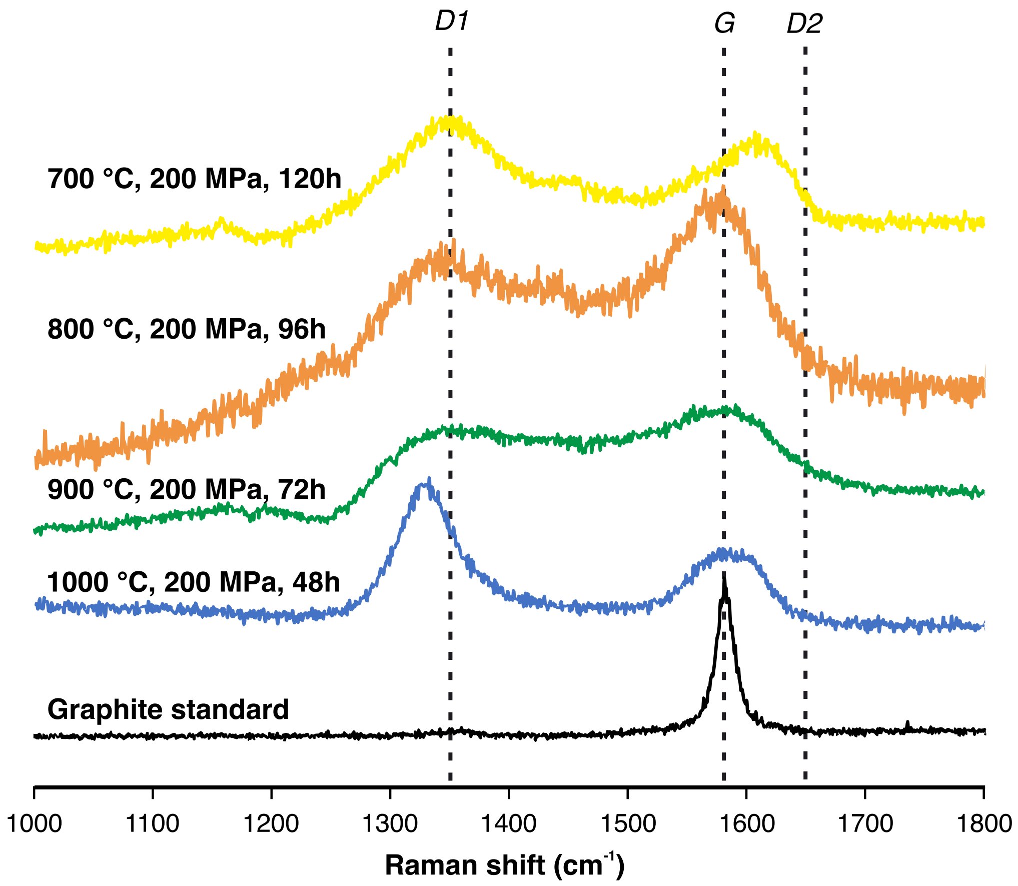

Figure S3. Stacked unprocessed Raman spectra of graphite of the 700 °C, 800 °C, 900 °C, and 1000 °C experiments as well as of the graphite standard material. The position of the *G*-band is shown at 1580 cm^{-1}, and the structural disorder bands *D1* and *D2* are shown at 1350 cm^{-1} and 1650 cm^{-1}, respectively (see, e.g., Henry et al.[18]). The experiment spectra have high background to peak ratio due to sample fluorescence.

The ferrogedrite spectrum was identified in the products of the 700 °C experiment. The measured spectrum is compared to a reference ferrogedrite, chamosite (Fe-rich chlorite), and clinochlore (Mg-rich chlorite) spectra obtained from the RRUFF database[4] in Fig. S4. The measured spectrum shows the characteristic ferrogedrite Raman shift peaks at ~400 cm^{-1}, ~530 cm^{-}

$^{-1}$, and ~650 cm^{-1}, although the characteristic peak at ~530 cm^{-1} is slightly off positioned at ~520 cm^{-1} (Fig. S4). The lower intensity characteristic spectra at ~700 cm^{-1} and ~1000 cm^{-1} are missing from the measured spectrum probably due to the increasingly higher background signal towards larger Raman shift (Fig. S4). We checked the Raman spectra of all the minerals (RRUFF database) identified in the starting material (quartz, chlorite, muscovite, albite, orthoclase, rutile, titanite, apatite, and pyrite) and of the additional minerals identified in the 800 °C experiment run products (cordierite, ferrogedrite, magnetite, ilmenite, and pyrrhotite) to verify that the spectrum represents ferrogedrite and not a combination of other minerals.

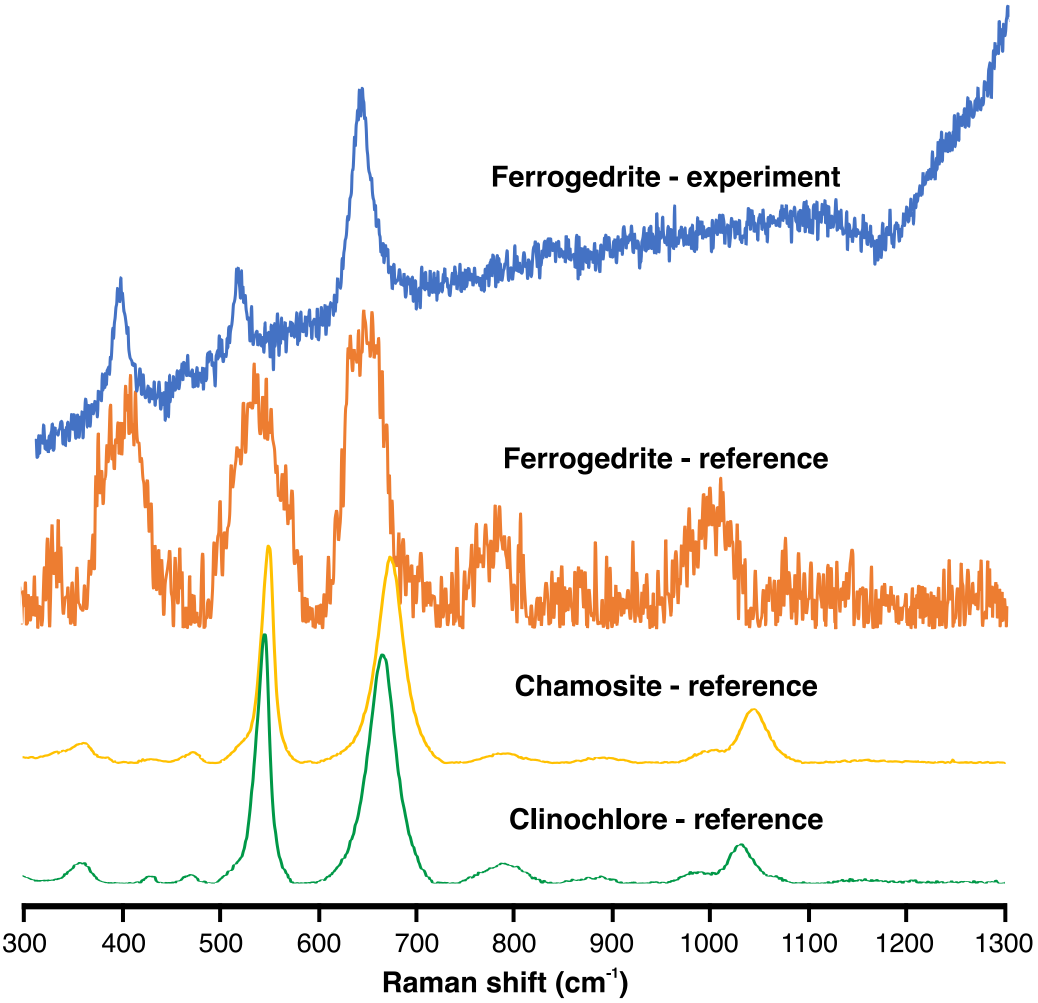

Figure S4. Stacked unprocessed Raman spectra of ferrogedrite in the 700 °C experiment products and the reference spectra for ferrogedrite, chamosite (Fe-rich chlorite), and clinochlore (Mg-rich chlorite). The reference spectra are from the RRUFF database[4].

SUPPLEMENTARY DISCUSSION

Graphite stability at the experimental conditions

In order to test whether the graphitic carbon was stable at the experimental conditions or if it precipitated after quenching, we conducted thermodynamic modeling for graphite stability with the PerpleX software (version 6.8.9[5]). We used the FLUIDS protocol with the modified Redlich-Kwong equation of state[6] to define the COHS fluid composition in a graphite-saturated system. The fS_2 for each experiment was defined with the method 2 of Mengason et al.[7] using the average pyrrhotite composition of each experiment (Supplementary Data 4–7). The inferred experimental logfS_2 are: -3.8 at 700 °C, -2.1 at 800 °C, -0.6 at 900 °C, and -0.9 at 1000 °C (Fig. S5). Pyrrhotite at the 900 °C and 1000 °C experiments, contains Ni, of which effect is not accounted for fS_2 in the Mengason et al.[7] method. We calculated the fS_2 for the models by approximating the effect of Ni to be similar with Cu. We also tested calculating the fS_2 with Ni-free pyrrhotite formulae, which resulted in 0.2 and 0.4 log units lower fS_2 for the 900 °C and 1000 °C fluids, respectively (Fig. S5). This difference in fS_2 does not affect the interpretation of graphite-stability in our experiments.

The modelled graphite-saturated COHS fluid compositions for each experiment are shown projected on the COH ternary (Fig. S5). We cannot accurately define how much H_2O silicate dehydration contributes to the fluid phase in the experiments, hence we show the projected fluid compositions at different kerogen-H_2O mixes on the red line in Fig. S5. The sample bulk composition in COH ternary is at the point where 100 % of the H_2O in the sample is mixed with the kerogen composition (Fig. S5). For the 700 °C, 800 °C, and 900 °C experiments, all the compositions plot in the graphite-saturated field (Fig. S5), which indicates graphite stability in these experiments. Graphite stability is also realized for the 1000 °C experiment, if less than ~90 mol% of H_2O (that is total H_2O in the black shale sample material) is hosted by the fluid (Fig. S5). Silicate

melt is the main phase at the 1000 °C experiment and is likely able to dissolve most of the H_2O, hence graphite was highly likely a stable phase.

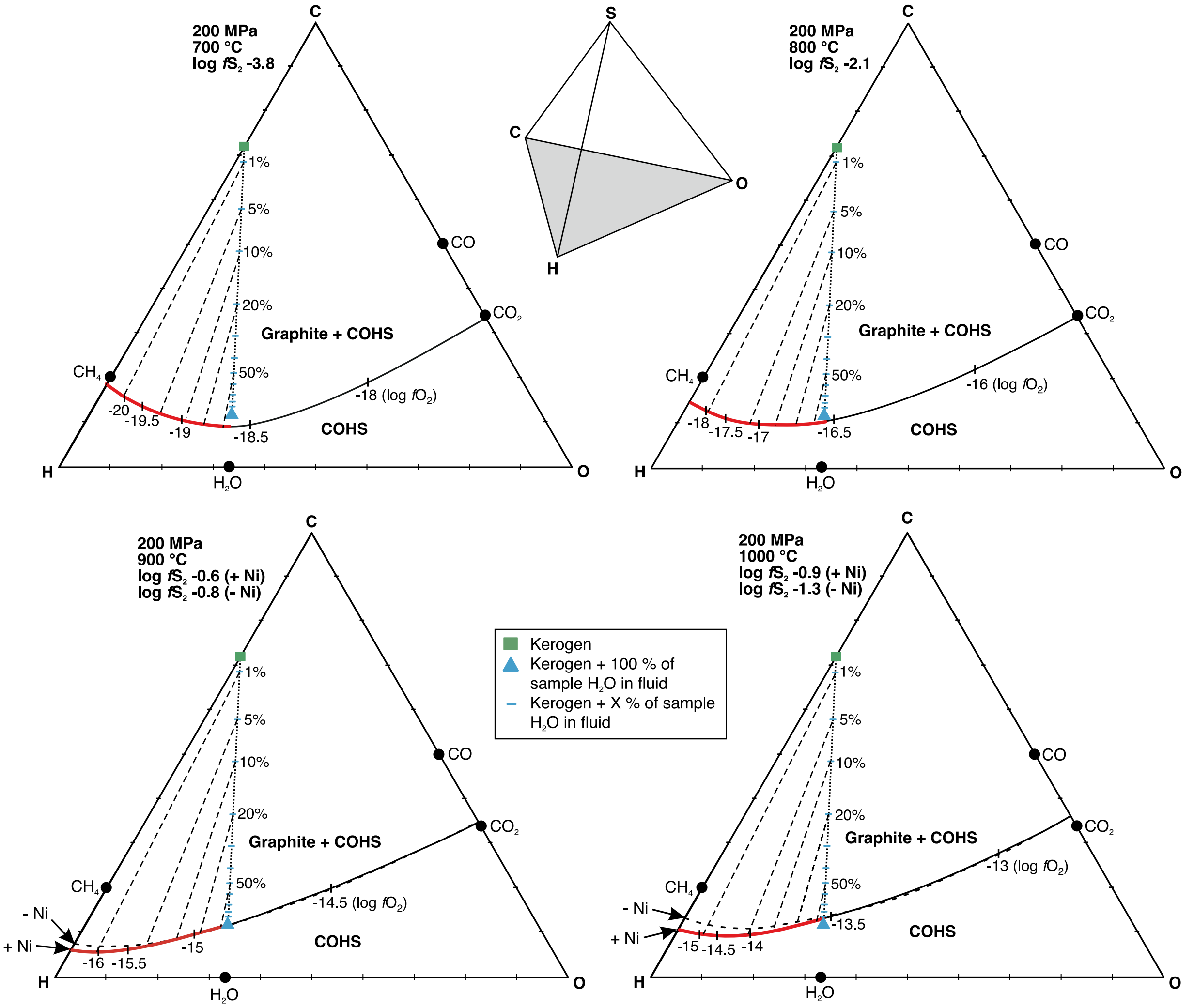

Figure S5. The PerpleX COHS fluid models for the isobaric (200 MPa) experiments at 700 °C, 800 °C, 900 °C, and 1000 °C projected on COH ternaries (see the text for modeling details). The fS_2 was constrained with the average pyrrhotite composition from each experiment using the method 2 of Mengason et al.[7]. At the 900 °C and 1000 °C experiments, pyrrhotite contains Ni, which was treated in the calculation either as Cu (+ Ni) or removed from the pyrrhotite formula (- Ni). The composition of the graphite-saturated fluid on the COH ternary lies on the red line and is constrained by the amount of H_2O in the fluid. The blue triangle shows the composition if all the H_2O in the sample material is available to the fluid system. The black stippled lines show where the fluid composition lies on the red line when different amounts (mol%) of H_2O is available to the fluid.

Analysis of Fe-S-Cu diffusion in the experiment capsules using the LA-ICP-MS data

Noble metal capsules have been reported to exchange Fe, S, and Cu with samples at various experimental conditions[8–12]. We measured our experiment capsules with LA-ICP-MS to verify that Fe, S, and Cu did not diffuse from the sample material into the capsule or vice versa in amounts that would compromise our mass balance considerations. The detailed data can be found in Supplementary Data 9. The LA-ICP-MS measurements were conducted on the capsule quarters detached from the sample for the analysis of the interior walls (Fig. S2) and on the capsule quarters attached to epoxy for the analysis of the capsule cross-sections (Fig. S2). Before the interior wall measurements, the empty quarter capsules were washed with distilled H_2O in ultrasonic bath to remove possible loose remnants of the black shale sample material. As the interior walls were in direct contact with the sample material during the experiments, they should show the different concentrations of Fe, S, and Cu, compared to the cross-sections (which represent the original composition of the capsule material) if diffusion of these elements occurred. Here we concentrate only on the standardization and analysis of Fe, S, and Cu and their implications.

For standardization, we used the average Au content of 15 $Au_{90}Pd_{10}$ capsules made from the same batch of capsule raw material as used in our experiments. The compositions of the capsules used as standards were measured with a JEOL-JXA 8200 EMPA at the Institute of Geochemistry and Petrology, ETH Zürich. The average Au content used as the internal standard is 90.9 wt.% ($1\sigma = 0.14$, n = 255)[13]. The average Pd content of these capsules is 9.1 wt.% ($1\sigma = 0.14$, n = 255)[13]. With the Au concentration of 90.9 wt.% as the internal standardization value, our LA-ICP-MS measurements show Pd concentrations of 6.0–6.8 wt.% and the total concentrations are in the range 97.3–98.2 wt.% (Supplementary Data 9).

The low total concentrations of our measurements can mean that the Au/Pd of our capsules is slightly higher than in the capsules used as standards, but this is unlikely since the capsule

material comes from the same batch of $Au_{90}Pd_{10}$ raw material that shows only minor variation in Au/Pd. Alternatively, this can mean that the instrumental accuracy for Pd, and possibly for the other elements as well, is poor for metallic samples. Therefore, it is important to note that our purpose here is not to provide absolute element concentrations but qualitatively define whether elements from the sample diffused into the capsule during experiments. For this, instrument precision is more important than accuracy.

The cross-section measurements revealed that in addition to Au and Pd, the capsules contain Cu (~1500–1700 ppm in the measurements), S (~300–600 ppm), and Fe (<100 ppm) as impurities (Supplementary Data 9). The Cu, S, and Fe impurities observed in the cross section measurements far from the sample contact must result from manufacturing of the capsule material (Fig. S2). This was further verified by measuring one additional capsule made from the same batch of the $Au_{90}Pd_{10}$ capsule material, but which contained a Cu and S-free synthetic sample produced with laboratory quality raw materials[14]. The LA-ICP-MS measurements of this reference capsule show similar amounts of the considered elements.

All the interior wall measurements contained 39–196 ppm Fe and one of the cross-section measurements 38 ppm Fe (Supplementary Data 9). In many of the interior measurements, the counts per second for Fe are higher in the beginning of the measurement, which shows that the Fe enrichment is higher close to the sample contact (Fig. S6). The total measured depth, i.e., the depth of the ablation pit, is ~20 μm and the higher Fe counts are typically present within the first ~20–30 seconds of the 50 second measurement (Fig. S6). This indicates that the diffusion distance for Fe is < 20 μm in all of the experiments and consequently the mass of the portion of the capsule that acquired Fe from the sample is smaller (< 5 mg) than the mass of the sample material (15–20 mg). As the black shale sample material contains 6.9 wt.% $Fe_2O_3^{total}$ (Supplementary Data 1), the Fe losses to the capsules are in the range of 0.003–0.008 wt.% relative to the total Fe in the sample, which is considered negligibly small.

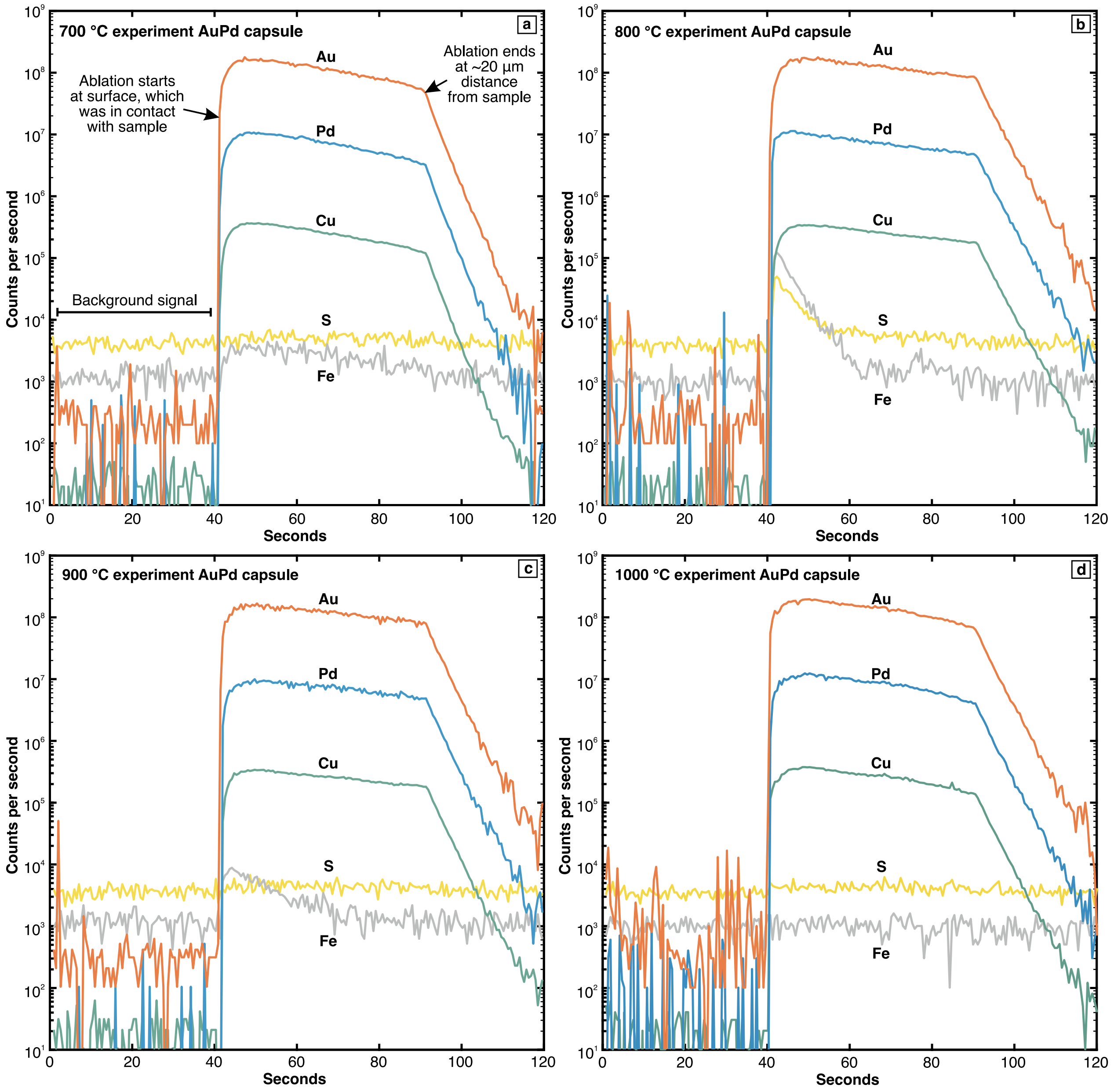

Figure S6. Representative LA-ICP-MS measurement spectra for the interior walls of the 200 MPa AuPd capsules used at a 700 °C, b 800 °C, c 900 °C, and d 1000 °C experiments. The 700 °C and 1000 °C capsules show a steady decrease in all measured element concentrations as the ablation depth increases after the start of the measurement at ~40 seconds, and then a sudden decrease, when the measurement ends between 90–100 seconds. The 800 °C and 900 °C capsule measurements are similar in the case of Au, Pd, and Cu, but Fe, and S in the case of 800 °C, show sudden increase and subsequent decrease within the first ~20 seconds of the measurement.

The average S and Cu contents in the capsule interiors and cross-sections are mainly uniform within 1σ error confidence level (Fig. S7). The raw counts per second data of the LA-ICP-MS measurements show constant differences between Cu, Pd, and Au in of all the interior wall measurements (Fig. S6). This indicates that there is no exchange (depletion or enrichment) of Cu at the portion of the capsule that was in direct contact with the black shale sample material during the experiments relative to the portion of the capsule that was at ~20 μm distance from the sample. For S, only the 800 °C experiment capsule shows elevated contents in the interior wall (Figs. S6b and S7b). The difference between the average S contents of the capsule cross-section and interior in the 800 °C experiment capsule is ~280 ppm. The elevated S content at the 800 °C experiment is related to the first ~20 seconds of the ablation, which indicates that the diffusion distance of S to the capsule is < 20 μm as in the case of Fe (Fig. S6). As the mass of the capsule with increased S content is considerably smaller (< 5 mg) than the mass of the sample material (15–20 mg). With 3690 ppm S in the black shale sample (Supplementary Data 1), the loss of ~1.7 wt.% relative to the total S, i.e., ~60 ppm, from the sample material in the 800 °C experiment is considered negligibly small.

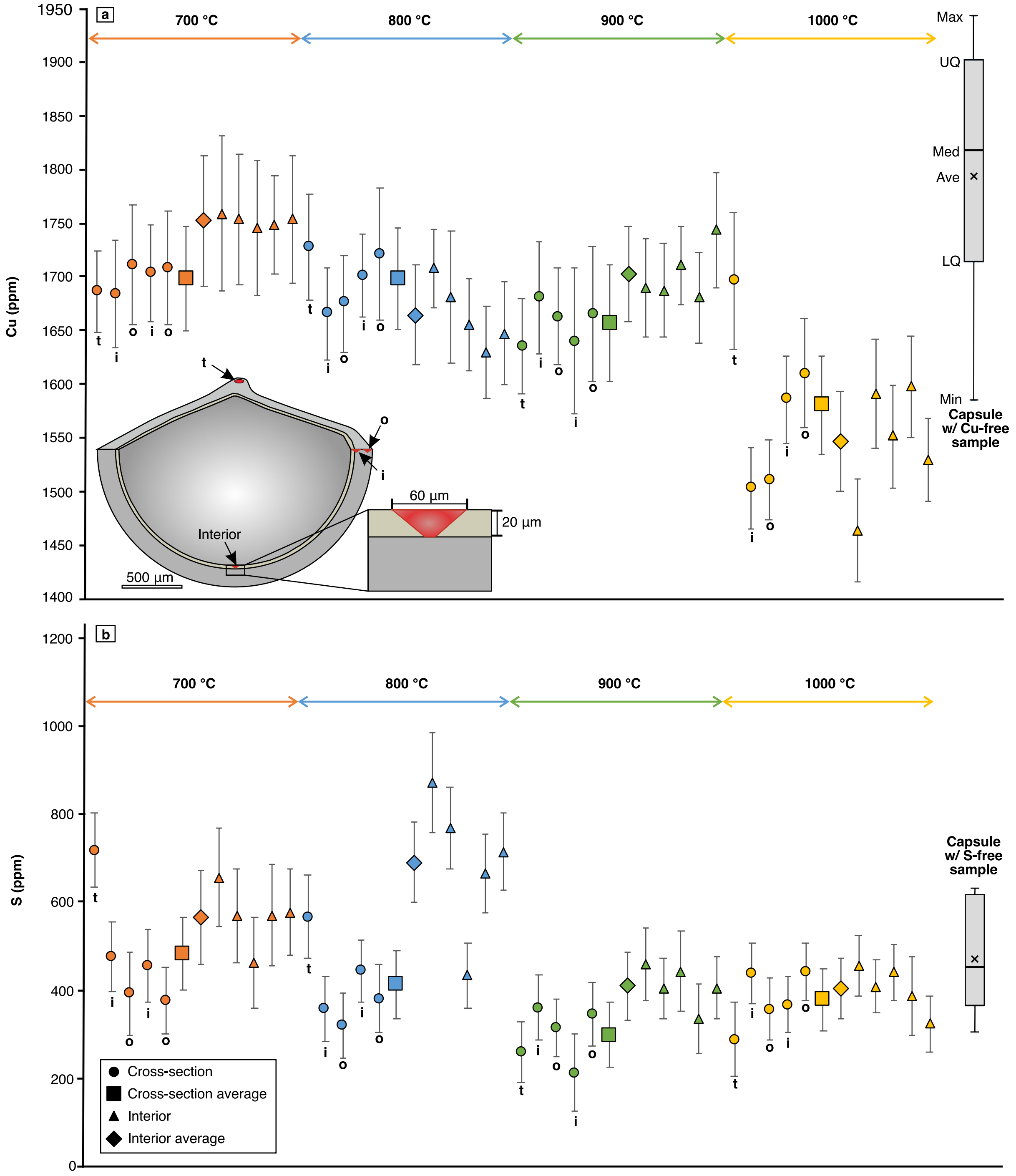

Figure S7. a Copper and b sulphur contents of the $Au_{90}Pd_{10}$ capsules used in the 200 MPa experiments at 700°C (orange), 800 °C (blue), 900 °C (green), and 1000 °C (yellow) as well as in an experiment with Cu and S-free synthetic sample material (box plots). The cross-section compositions (circles) have been measured from the cross-section of the capsule wall and are considered as free of any sulphur and copper from the sample material. The interior compositions (triangles) represent measurements from the inside walls of the capsules, which were directly in touch with the sample material. The black bars for the individual measurements represent 1σ errors and for the average measurements they represent averages of the 1σ errors of the individual measurements. The box plots indicate average composition (ave), median (med), lower quartile (LQ), upper quartile (UQ), and the whole compositional range (min and max). See the Fig. S2 caption for explanation of the schematic illustration of the measurement distribution shown in the bottom of the copper concentration diagram.

Because our estimation of the Cu content in the fluid at the 700 °C experiment is purely based on the mass balance calculations, a rigorous consideration of the possibility to detect Cu diffusion from the sample into the capsule material is required. The Cu content of the sample material is an order of magnitude smaller (125 ppm) relative to the Cu content of AuPd capsule material (~1500–1700 ppm), hence detecting increase in capsule Cu content requires higher precision compared to Fe and S. The relative masses of the sample material and the portion of the capsule that could have acquired Cu via diffusion determine how much the Cu content of the capsule could increase. To estimate the distance of Cu diffusion into the capsule at the 700 °C experiment, we calculated the capsule volume that could have gained Cu by diffusion from the sample during the experiment using the Arrhenius law:

$$D = A \times e^{-Q/RT}, \qquad (1)$$

where D is the diffusion rate, A is the pre-exponential factor, Q is the activation energy, R is the universal gas constant, and T is the temperature in Kelvins. For the calculation, we used Cu in Au pre-exponential factor of $10^{-2.92}$ cm^2/s and activation energy of 28.5 kcal/mol[15]. The diffusion distance was calculated with the relation

$$\sigma = \sqrt{2D \times t}, \qquad (2)$$

where σ is the diffusion distance and t is the time, i.e., the duration of the experiment. Accordingly, Cu diffusion to the capsule during the 120h experiment at 700 °C is ~20 μm, which coincidentally is about the same as the depth of the laser ablation pits.

Based on the approximated diffusion distance, we calculated that the potential amount of sample-derived Cu contamination within the diffusion volume of the capsule would be ~300 ppm,

given that the Cu distributed equally within the reacted capsule volume and all of the Cu (~70 wt.% of the total Cu) that is missing from the solid sulphides in the sample was assimilated by the capsule. Note that in reality, Cu should be concentrated close to the capsule-sample interface with gradual decrease towards the maximum diffusion distance as observed in the case of Fe and S at the capsule used in the 800 °C experiment (Fig. S6b).

The precision of the LA-ICP-MS measurements for Cu is high enough (1σ errors for Cu are 35–70 ppm) to detect the possible Cu exchange between the sample to the capsule. The Cu compositions of the inner wall of the capsule and the cross-section are nevertheless same within uncertainty (Fig. S7) and the Cu concentration within the whole capsule volume is constant relative to Au and Pd (Fig. S6). Hence we conclude that no significant Cu diffusion occurred during the 700 °C experiment and that the mass balance calculations considering Cu distribution are valid.

Estimating the mass of solid sulphides in the 700 °C experiment run products

The second mass balance calculation presented in the following section is used to calculate how much S and Cu are hosted by the solid sulphides and the fluid. The calculation is based on the assumption that the mass of Fe bound to the solid sulphides is constant between the starting material and the 700 °C experiment products. This assumption was tested by estimating the mass of solid sulphides in the 700 °C experiment products by analyzing a BSE image of the 700 °C experiment (Fig. S8) using ImageJ image analysis software (version 1.53a).

With the exception of thin rims around rare titanite and rutile (see Fig. 1a in the main text), sulphides are the only bright phases in the BSE image (Fig. S8) and can thus be identified based on color. In order to determine the relative area of the sulphides, the capsule walls were excluded from the BSE map after which the image was converted to 8-bit format. By defining a threshold value that determines the areas with white (representing sulphides) and darker color (representing other phases), the BSE image was converted to a binary color (black and white) image (Fig. S8). The

relative areas of white and black in the binary image define the areas of sulphides and other phases, respectively. This procedure was repeated with four different threshold values. One large sample area with four smaller subsample areas (within the larger area) were investigated from all four the binary images to account for heterogeneity in the sulphide distribution (Fig. S8).

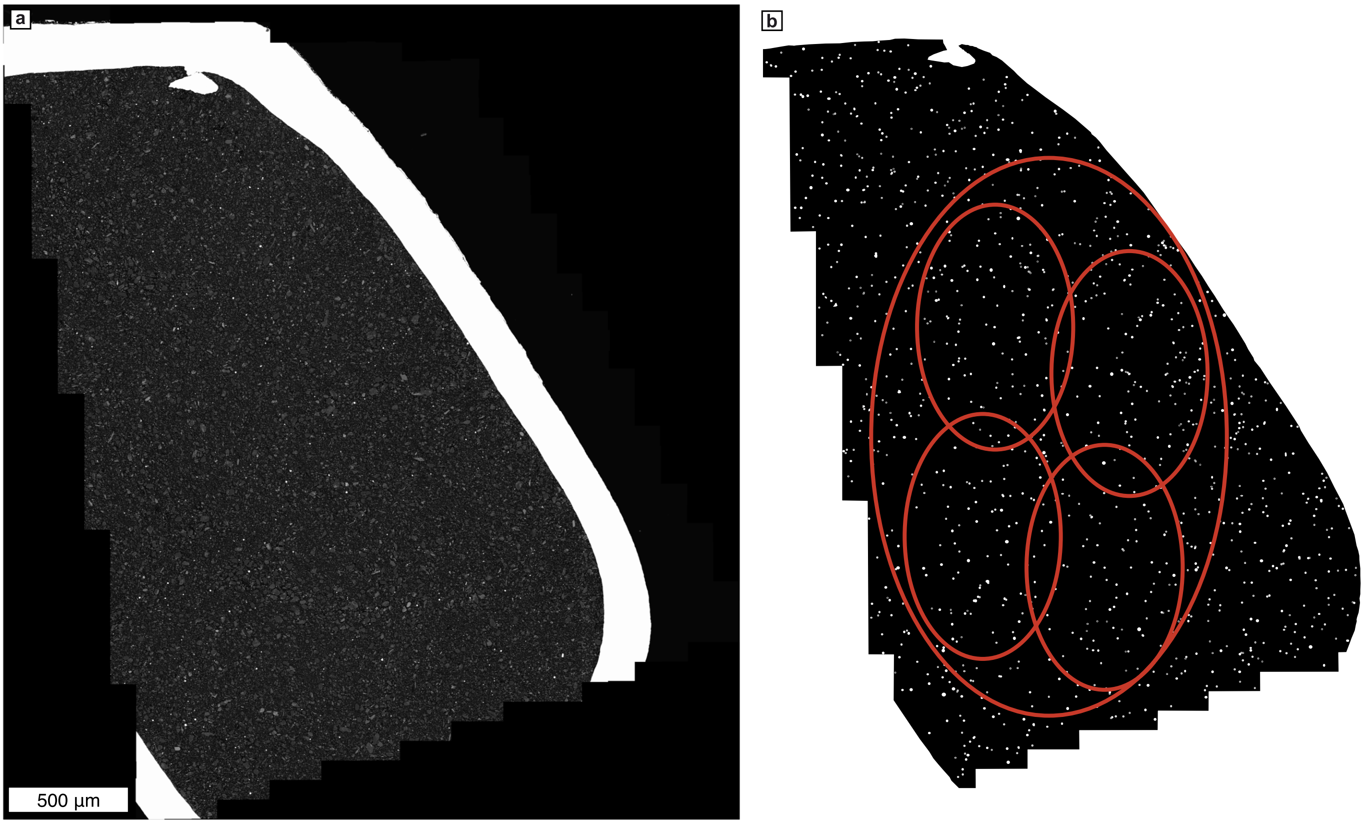

Figure S8. a Back-scattered electron image map of the 700 °C experiment and b an example of the binary color map with sulphides shown in white and the rest of the material in black. The red ellipses indicate the different areas, where the relative areas for sulphides and silicates were measured.

The area estimates for pyrrhotite range from 0.26 to 0.50% depending on the color threshold and the sampled area. To calculate the relative masses of pyrrhotite and the rest of the sample material, we used densities of 4.61 g/cm^3 and 2.8 g/cm^3, respectively, the latter representing the average density of the not contact metamorphosed Virginia Formation black shale[16]. Based on these constraints, pyrrhotite constitutes 0.46–0.82 wt.% of the sample. Constant iron content in solid sulphides between the starting material and the 700 °C products is a requirement for the second mass balance calculation. This is realized at 0.52 wt.% pyrrhotite, which is within the range of the estimations.

Note that with the whole range of estimated pyrrhotite masses, certain amount of the total Cu and S have been extracted from the pyrrhotite. This is compatible with the suggested fluid activity.

Mass balance calculations

Mass balance calculation 1

The chemical data used in the mass balance calculations 1 and 2 are explained in the methods section of the main text and the used values can be found in the Supplementary Data 1, 3, and 4. Pyrite to chalcopyrite ratio in the starting material (VF-BS1) is needed to constrain the bulk sulphide composition in the experiment starting material. As the spatial sulphide distribution is highly heterogeneous on thin section scale, the ratio is calculated based on the assumption that all S and Cu are in sulphides and that the amount of sulphides other than pyrite and chalcopyrite (e.g. sphalerite and galena) is negligible as suggested by petrographic observation and FE-SEM EDS measurements of the starting materials.

Average S concentration in pyrite (Py_S) = 54.5 wt.% (FE-SEM EDS)
Average Cu concentration in pyrite (Py_{Cu}) = 0.1 wt.% (FE-SEM EDS)
Average S concentration in chalcopyrite (Cpy_S) = 33.9 wt.% (FE-SEM EDS)
Average Cu concentration in chalcopyrite (Cpy_{Cu}) = 35.9 wt.% (FE-SEM EDS)
S concentration in whole rock (WR_S) = 0.369 wt.% (Infrared absorption)
Cu concentration in whole rock (WR_{Cu}) = 0.0125 wt.% (ICP-MS)
Fraction of pyrite in whole rock = x
Fraction of chalcopyrite in whole rock = y
Proportion of pyrite from total sulphides = A
Proportion of chalcopyrite from total sulphides = B

The conditions that must be realized are

$$Py_S \times x + Cpy_S \times y = WR_S \qquad (1.1)$$

$$Py_{Cu} \times x + Cpy_{Cu} \times y = WR_{Cu} \qquad (1.2)$$

Rearranging equation (MB1.1) gives

$$y = \frac{WR_S - Py_S \times x}{Cpy_S} \qquad (1.3)$$

Substituting *y* from equation (1.2) by right hand side of equation (1.3) gives

$$Py_{Cu} \times x + Cpy_{Cu} \times \frac{WR_S - Py_S \times x}{Cpy_S} = WR_{Cu} \tag{1.4}$$

Rearranging equation (1.4) allows to calculate *x*

$$x = \frac{WR_{Cu} \times Cpy_S - Cpy_{Cu} \times WR_S}{Cpy_S \times Py_{Cu} - Cpy_{Cu} \times Py_S} \approx 6.6 \times 10^{-3} \tag{1.5}$$

Substituting *x* from equation (1.1) by equation (1.5) gives

$$\frac{Py_S \times (WR_{Cu} \times Cpy_S - Cpy_{Cu} \times WR_S)}{Cpy_S \times Py_{Cu} - Cpy_{Cu} \times Py_S} + Cpy_S \times y = WR_S \tag{1.6}$$

Rearranging equation (1.5) allows to calculate *y*

$$y = \frac{WR_S}{Cpy_S} - \frac{Py_S \times (WR_{Cu} \times Cpy_S - Cpy_{Cu} \times WR_S)}{Cpy_S \times (Cpy_S \times Py_{Cu} - Cpy_{Cu} \times Py_S)} \approx 3.3 \times 10^{-4} \tag{1.7}$$

Calculating *A* from the *x* by:

$$A = \frac{x \times 100wt.\%}{x+y} \approx 95.2\% \tag{1.8}$$

Calculating *B* from the y by:

$$B = \frac{y \times 100wt.\%}{x+y} \approx 4.8\% \tag{1.9}$$

Mass balance calculation 2

In order to calculate how much S and Cu are mobilized by the fluid phase in the 700 °C experiment, the minimum masses of S and Cu that partition from solid sulphides into the fluid phase are calculated using the Fe-Cu-S ternary system. This requires the assumption that all Fe stays in the solid sulphides, which is compatible with the range of pyrrhotite volume estimates (see the previous section). If some Fe partitions into the fluid as well, the mass of fluid increases and mass of solid sulphides decreases.

Bulk sulphide (BS) composition of the experiment starting material (VF-BS1) is first calculated in the Fe-Cu-S ternary system by multiplying the measured pyrite and chalcopyrite compositions of the starting material with their relative proportions calculated with the mass balance calculation 1.

Average Fe concentration in pyrite (Py_{Fe}) = 45.4 wt.% (FE-SEM EDS)
Average Cu concentration in pyrite (Py_{Cu}) = 0.1 wt.% (FE-SEM EDS)
Average S concentration in pyrite (Py_{S}) = 54.5 wt.% (FE-SEM EDS)
Average Fe concentration in chalcopyrite (Cpy_{Fe}) = 30.2 wt.% (FE-SEM EDS)
Average Cu concentration in chalcopyrite (Cpy_{Cu}) = 33.9 wt.% (FE-SEM EDS)
Average S concentration in chalcopyrite (Cpy_{S}) = 35.9 wt.% (FE-SEM EDS)
Proportion of pyrite from total sulphides ($Py_{wt.\%}$)= 95 wt.% (From 1.8)
Proportion of chalcopyrite from total sulphides ($Cpy_{wt.\%}$)= 5 wt.% (From 1.9)
Bulk sulphide Fe concentration = BS_{Fe}
Bulk sulphide Cu concentration = BS_{Cu}
Bulk sulphide S concentration = BS_{S}

Fe concentration of the BS is calculated by

$$BS_{Fe} = Py_{Fe} \times \mathrm{Py}_{wt.\%} + Cpy_{Fe} \times Cpy_{wt.\%} \approx 44.7\, wt.\% \quad (2.1)$$

Cu concentration of the BS is calculated by

$$BS_{Cu} = Py_{Cu} \times \mathrm{Py}_{wt.\%} + Cpy_{Cu} \times Cpy_{wt.\%} \approx 1.7\, wt.\% \quad (2.2)$$

S concentration of the BS is calculated by

$$BS_{S} = Py_{S} \times \mathrm{Py}_{wt.\%} + Cpy_{S} \times Cpy_{wt.\%} \approx 53.6\, wt.\% \quad (2.3)$$

The minimum mass of fluid (in the Fe-Cu-S ternary system) that forms can be calculated by the assumption that all Fe (BS_{Fe}) stays in the residual solid pyrrhotite.

Average Fe concentration in pyrrhotite (Po_{Fe}) = 59.3 wt.% (FE-SEM EDS)
Assumed Fe concentration in fluid (F_{Fe}) = 0 wt.%
Mass of pyrrhotite = M_{Po}
Mass of fluid = M_{F}

First, the amount of residual solid is calculated by

$$M_{Po} = \frac{BS_{Fe} - M_F \times F_{Fe}}{Po_{Fe}} \quad (2.4)$$

With the assumption that F_{Fe} is 0 wt.%, the equation can be modified to

$$M_{Po} = \frac{BS_{Fe}}{Po_{Fe}} \approx 75.3\, wt.\% \quad (2.5)$$

The M_F in wt.% can then be calculated by

$$M_F = 1 - M_{Po} \times 100\, wt.\% \approx 24.7\, \mathrm{wt.}\% \quad (2.6)$$

As the relative amounts of M_{Po} and M_F are known, the fluid S and Cu compositions in the Fe-Cu-S ternary system can be calculated by mass balance.

Average Cu concentration in pyrrhotite (Po_{Cu}) = 0.9 wt.% (FE-SEM EDS)
Average S concentration in pyrrhotite (Po_S) = 39.8 wt.% (FE-SEM EDS)
Calculated Cu concentration in BS (BS_{Cu}) = 1.8 wt.% (From 2.2)
Calculated S concentration in BS (BS_S) = 53. 5wt.% (From 2.3)
Mass of pyrrhotite (M_{Po}) = 75 wt.% (From 2.5)
Mass of fluid (M_F) = 25 wt.% (From 2.6)
Proportion of Cu in fluid = F_{Cu}
Proportion of S in fluid = F_S
wt.% of total Cu in fluid = F_{Cut}
wt.% of total S in fluid = F_{St}

The F_{Cu} can be calculated from the following equation:

$$F_{Cu} \times M_F + Po_{Cu} \times M_{Po} = BS_{Cu} \quad (2.7)$$

Rearranging the equation (2.7) gives

$$F_{Cu} = \frac{BS_{Cu} - Po_{Cu} \times M_{Po}}{M_F} \approx 4.21 \quad (2.8)$$

The F_{Cut} can be calculated by

$$F_{Cut} = \frac{F_{Cu} \times M_F}{BS_{Cu}} \times 100\, wt.\% \approx 60.5\% \quad (2.9)$$

The F_S can be calculated from the following equation:

$$F_S \times M_F + Po_S \times M_{Po} = BS_S \quad (2.10)$$

Rearranging the equation (2.10) gives

$$F_S = \frac{BS_S - Po_S \times M_{Po}}{M_F} \approx 95.8 \quad (2.11)$$

The F_{St} can be calculated by

$$F_{St} = \frac{F_S \times M_F}{BS_S} \times 100\, wt.\% \approx 44.2\% \quad (2.12)$$

SUPPLEMENTARY REFERENCES

1. Luque, F. J., Pasteris, J. D., Wopenka, B, Rodas, M., and Barrenechea, J. F. Natural fluid-deposited graphite: mineralogical characteristics and mechanisms of formation. *American Journal of Science* **298**, 471–498 (1998).
2. Pasteris, J. D. & Chou, I-M. Fluid-deposited graphitic inclusions in quartz: comparison between KTB (German Continental Deep-Drilling) core samples and artificially reequilibrated natural inclusions. *Geochimica et Cosmochimica Acta* **62**, 109–122 (1998).
3. Kueter, N., Lilley, M. D., Schmidt, M. W. & Bernasconi, S. M. Experimental carbonatite/graphite carbon isotope fractionation and carbonate/graphite thermometry. *Geochimica et Cosmochimica Acta* **253**, 290–306 (2019).
4. Lafuente, B., Downs, R. T., Yang, H. & Stone, N. in Highlights in Mineralogical Crystallography (eds Armbruster, T. & Danisi, R. M.) 1–30 (Berlin, Germany, W. De Gruyter, 2015).
5. Connolly, J. A. D. The geodynamic equations of state: what and how. *Geochemistry, Geophysics, Geosystems* **10**, 1–19 (2009).
6. Connolly, J. A. D. & Cesare, B. C-O-H-S fluid composition and oxygen fugacity in graphitic metapelites. *Journal of Metamorphic Geology* **11**, 379–388 (1993).
7. Mengason, M. J., Piccoli, P. M. & Candela, P. An evaluation of the effect of copper on the estimation of sulfur fugacity (fS_2) from pyrrhotite composition. *Economic Geology* **105**, 1163–1169 (2010).
8. Urabe, T. Aluminous granite as a source magma of hydrothermal ore deposits: an experimental study. *Economic Geology* **80**, 148–157 (1985).
9. Adam, J. & Green, T. Trace element partitioning between mica- and amphibole-bearing garnet lherzolite and hydrous basanitic melts: 1. Experimental results and the investigation

of controls on partitioning behaviour. *Contributions to Mineralogy and Petrology* **152**, 1–17 (2006).

10. Fellows, S. A. & Canil, D. Experimental study of the partitioning of Cu during partial melting of Earth's mantle. *Earth and Planetary Science Letters* **337–338**, 133–143 (2012).
11. Zajacs, Z., Seo, J. H., Candela, P. A., Piccoli, P. M. & Tossell, J. A. The solubility of copper in high-temperature magmatic vapors: A quest for the significance of various chloride and sulfide complexes. *Geochimica et Cosmochimica Acta* **75**, 2811–2827 (2011).
12. Wang, J., Xiong, X., Zhang, L. & Takahashi, E. Element loss to platinum capsules in high-temperature–pressure experiments. *American Mineralogist* **105**, 1593–1597 (2020).
13. Marxer, F. Polybaric fractional crystallization of arc magmas – an experimental study. Zürich: Doctoral Thesis, ETH Zürich, 1–369 (2021).
14. Manoochehri, S. & Schmidt, M. W. Settling and compaction of chromite cumulates employing a centrifuging piston cylinder and application to layered mafic intrusions. *Contributions to Mineralogy and Petrology* **168**, 1–20 (2014).
15. Kubaschewski, O. The diffusion rates of some metals in copper, silver, and gold. *Transactions of the Faraday Society* **46**, 713–722 (1950).
16. Rao, B. V. & Ripley, E. M. Petrochemical studies of the Dunka Road Cu-Ni Deposit, Duluth Complex, Minnesota. *Economic Geology* **78**, 1222–1238 (1983).
17. Benkó, Z., Mogessie, A., Molnár, F., Hauck, S. A., Severson, M. J. & Ettinger, K. The influence of thermal differences and variation of Cl-F-OH ratios on Cu-Ni-PGE mineralization in the contact aureole of the South Kawishiwi Intrusion, Duluth Complex. *Geosciences* **8**, 1–35 (2018).
18. Henry, D. G., Jarvis, I., Gillmore, G. & Stepheson, M. Raman spectroscopy as a tool to determine the thermal maturity of organic matter: application to sedimentary, metamorphic and structural geology. *Earth-Science Reviews* **198**, 1–19 (2019).
