# Supplementary material for: Fluids as primary carriers of sulphur and copper in magmatic assimilation
Source: Nat Commun. 2021 Nov 16;12:6609. doi: 10.1038/s41467-021-26969-3 (PMC8595724; doi:10.1038/s41467-021-26969-3)
Supplement: Supplementary file 2 — Description of the Supplementary Data [file 41467_2021_26969_MOESM2_ESM.pdf]

**Description of the Supplementary Data files for the manuscript "*Fluids as primary carriers for sulphur and copper in magmatic assimilation*".**

Virtanen et al.

**Supplementary Data:**

**Supplementary Data 1**: Bulk composition of the black shale starting material VF-BS1 used in the experiments

**Supplementary Data 2**: Inferred kerogen H/C in the black shale starting material VF-BS1 used in the experiments

**Supplementary Data 3**: FE-SEM sulphide measurements from the starting material VF-BS1 used in the experiments

**Supplementary Data 4**: FE-SEM sulphide measurements from the 700 °C, 200 MPa experiment run products

**Supplementary Data 5**: FE-SEM sulphide measurements from the 800 °C, 200 MPa experiment run products

**Supplementary Data 6**: FE-SEM sulphide measurements from the 900 °C, 200 MPa experiment run products

**Supplementary Data 7**: FE-SEM sulphide measurements from the 1000 °C, 200 MPa experiment run products

**Supplementary Data 8**: FE-SEM sulphide measurements from the marcasite and chalcopyrite standards

**Supplementary Data 9**: LA-ICP-MS measurements from the capsules used in the black shale experiments and a capsule from an experiment with a synthetic sample
